# Living Rapid Research Needs Appraisal (RRNA) for Priority Diseases Protocol

**DOI:** 10.12688/wellcomeopenres.25003.1

**Published:** 2025-12-10

**Authors:** Marieke de Swart, Dijana Spasenoska, Eli Harriss, Vincent Cheng, Katrin Probyn, Gemma Villanueva, Thomas Mendy, Musawenkosi Ndlovu, Brian Buckley, Daniela Toale, Nicholas Henschke, Duduzile Ndwandwe, Alice Norton, Louise Sigfrid

**Affiliations:** 1 Policy and Practice Research Group, Pandemic Sciences Institute, Nuffield Department of Medicine, University of Oxford, Oxford, England, UK; 2 Health Care Libraries, University of Oxford Bodleian Libraries, Oxford, England, UK; 3 University of Bristol Medical School, Bristol, England, UK; 4 Cochrane Response, Cochrane, London, England, UK; 5 Cochrane South Africa, Tygerberg, Cape Town, South Africa

**Keywords:** Priority Diseases: Emerging Infections: Rapid Research Needs Appraisal: Evidence Gaps: Research Prioritisation

## Abstract

Systematic reviews require time and resources to conduct that might not be available in a rapidly developing context such as during new and (re-) emerging infectious disease outbreaks. As part of the Pandemic-PACT program at the Pandemic Sciences Institute at the University of Oxford we have, in collaboration with Cochrane Response and Cochrane South Africa, developed a "living" Rapid Research Needs Appraisal (RRNA) methodology. The aim is to develop a protocol for rapidly and robustly identify key evidence gaps across pre-defined research domains to inform research prioritisation and coordination in preparedness time and during outbreaks. The RRNA utilises modified systematic review methods, a global relay of reviewers, and systematic review software. The objective is to strengthen capability for carrying out RRNAs, and establish a baseline evidence platform for priority infectious diseases that can be readily updated as new evidence emerges, utilising an adaptable "living" RRNA protocol. This data will be of relevance to funders, researchers, clinical and public health stakeholders involved in management of infectious diseases of epidemic and pandemic potential.

## Plain language summary

This document presents a methodology for carrying out a rapid research needs appraisal (RRNA) to be able to rapidly and robustly identify existing published evidence on infectious diseases of epidemic and pandemic potential in preparedness time and in response to new infectious disease outbreaks. These findings will be important for identifying where there are gaps in our evidence base, e.g. on effective treatment and vaccines for different population groups such as children, adults, older people, pregnant women, or people with pre-existing illnesses. This data will be important for informing prioritisation of research to address these gaps, to generate the evidence to inform clinical and public health management to prevent infections, reduce risk of transmission, and improve patient and outbreak outcomes.

## Introduction

Systematic reviews require time and resources that might not be available in a rapidly developing context such as during a (re-) emerging infectious disease outbreak. Even rapid evidence reviews often take months to complete ^1, 2^. At the initial stages of an outbreak, researchers, policy makers and funders need to rapidly identify gaps in evidence and knowledge to inform and prioritise clinical and public health research responses. During outbreaks, the complexity associated with research prioritisation is compounded by the need for a rapid response. Experiences from outbreaks show that this takes time, and for protracted outbreaks priorities may change as the outbreak progresses and new evidence and needs are identified ^3^.

This document presents a methodology for establishing a platform of ‘living’ rapid research needs appraisal (RRNA). The aim is to rapidly and robustly identify gaps in evidence across pre-defined research domains for new and re-emerging diseases of epidemic and pandemic potential, to inform research prioritisation and coordination in preparedness time and during outbreaks. The RRNA can be pivoted to provide additional evidence from the identified records to inform clinical and public health strategies if and when required, and can be developed to a full systematic review. The protocol is designed in a modular approach, with flexibility to target RRNAs depending on the disease, context, and recognised need. We aim to establish a platform of RRNAs for infectious diseases prioritised for research and development by the World Health Organisation and international experts ^4^. The objectives are to provide a baseline of evidence highlighting key gaps across a set of pre-defined, key research domain categories, which can be readily updated as new evidence emerges during outbreaks. A secondary objective is to provide a repository of published evidence that can readily be pivoted to present existing published evidence to inform research and public health strategies. This data will be of relevance to researchers, funders and policy makers to inform research prioritisation, coordination and pre-positioning of research ready to be implemented at outbreak outset to strengthen our global preparedness for a coordinated, effective and equitable research response to outbreaks, to generate evidence to inform strategies to protect populations, and improve outcomes.

## Protocol

This protocol has been developed in collaboration with methodologists, information specialists, infectious disease researchers with experience in outbreak response, public health specialists and social scientists at the University of Oxford, in collaboration with experts from Cochrane Response and Cochrane South Africa, and in consultation with multidisciplinary external experts. It builds on a prototype protocol developed earlier ^5^, which has been expanded in scope and flexibility to create a living modular platform that can readily and flexibly be implemented in a targeted approach. The methodology is derived from systematic evidence review methodologies and modified to allow the rapid and robust identification of evidence-gaps on emerging infectious diseases during outbreaks. The protocol is developed following PRISMA-P guidelines. The systematic evidence search will be carried out by a health information specialist, and the screening of records and data extraction by reviewers based in different time-zones, who are trained in the methodology to ensure rigour and quality. The minimum number of systematic reviewers at each stage is laid out in detail in later sections and can be scaled up depending on the volume of evidence identified at the search stage. The use of a web-based systematic review software will enable scaling up of resources as required and will allow processes such as screening and data extraction to be carried out in parallel by multiple reviewers. Using a pre-programmed systematic review software facilitates standardisation of data extraction and quality assurance, as well as rapid initiation of an RRNA and update of an existing RRNA in response to outbreaks. This is an open-access protocol describing a method for rapidly and systematically identifying gaps in the evidence base to inform research prioritisation for implementation in any resourced settings.

It is developed to facilitate implementation in any resourced setting, and global collaboration across regional time zones for effectiveness, quality and applicability. In the interest of global preparedness and response, open-access data will be made available via the Pandemic PACT website ( https://www.pandemicpact.org/). The RRNA utilises modified systematic review methodologies and a global relay model for efficiency, regional expertise and quality ( Figure 1). The protocol methodology and management process is described in detail below.

**Figure 1.**
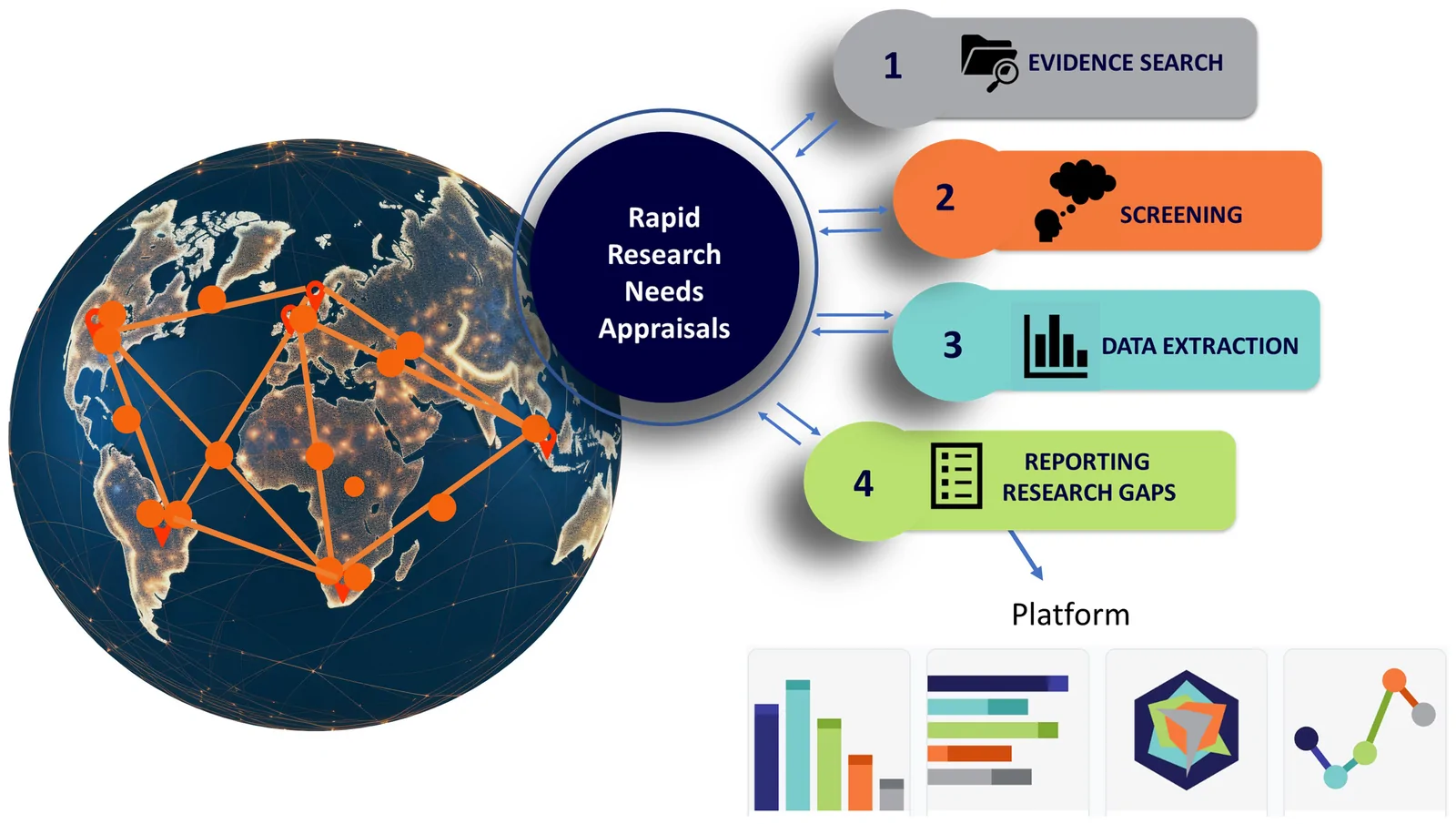
Rapid Research Needs Appraisal (RRNA) Process. **Image credit:** Cochrane South Africa.

### RRNA team roles and responsibilities

The decision to undertake an RRNA will be informed by available risk assessments, consultations with content experts and recognised need as appropriate depending on each outbreak. An RRNA will only be undertaken if after consultations it is deemed that there is insufficient evidence available to base an informed decision on clinical research priorities ( Figure 2). An RRNA steering group (SG) is responsible for informing the need and scope for an RRNA, and the capability and feasibility of implementing an RRNA in preparedness time and during outbreaks. The SG will review outbreak alerts and stakeholder requests and will decide when and if there is a need for an update. The SG will also take a decision on the need to target of the RRNA depending on each disease, disease family, outbreak and context, in consultation with the coordinating team and stakeholders as appropriate. The SG is responsible for resource management and for overseeing the project, scope, targeting, quality, analysis and dissemination of the findings. Moreover, deciding on need for and overseeing engagement and consultations with external stakeholders and experts if appropriate.

**Figure 2.**
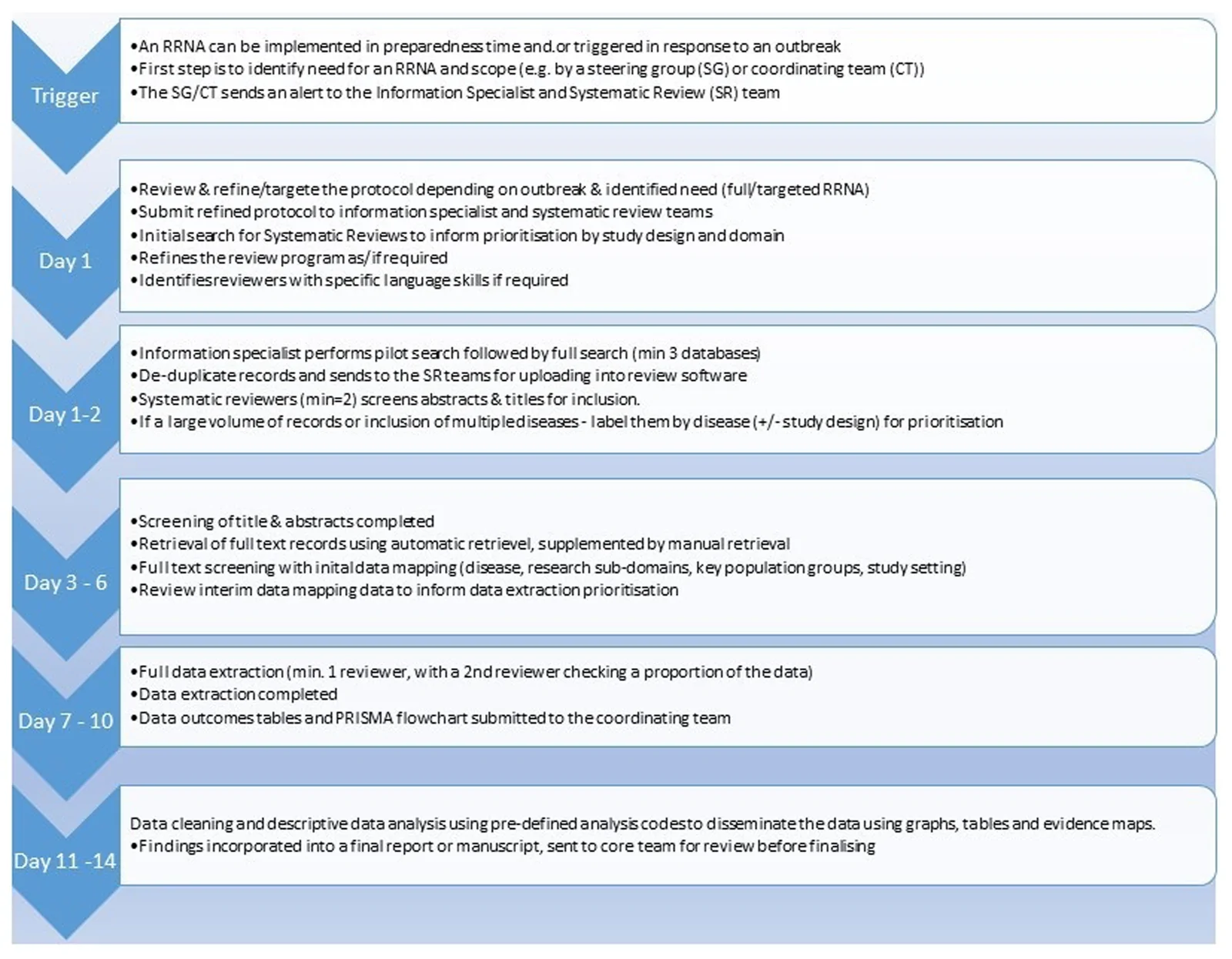
RRNA methodology overview.

**Abbreviations:** CT: coordinating team; RRNA: Rapid Research Needs Appraisal; SG: Steering Group; SR: systematic reviews

### Coordinating Team (CT)

The CT will be responsible for coordinating the process from notification of the need for a RRNA from the SG, through to completion ( Table 1). The SG and CT may be the same team depending on need and resources.

**Table 1.** Coordinating Team Responsibilities.

**Day 1–2: Preparation & alert phase**	To review the search strategy, pilot search results, available systematic reviews, and refinement of the protocol, and liaise with information specialist, reviewers and internal and external experts as appropriate to inform the need for any amendments to the protocol for each disease and outbreak context.
	To finalise amendments and targeting of the protocol.
	To submit the final protocol with any amendments clearly highlighted in track changes to the search specialist and systematic review team/s.
	To provide a brief overview of the disease with relevant background information.
**Day 2–10**	To retrieve full text articles not identified by EndNote’s automatic retrieval function and be on stand-by to provide clinical or content specific advice as required.
**Day 4–14**	To support screening of articles and data extraction.
	To monitor the progress and help find solutions to any unexpected issues.
**Day 6–10**	Full text screening and data extraction
**Day 10–14**	To collate, tidy and present the data outcome tables and notes from the systematic review team/s with gaps analysis in the final report and submit the report to the steering group and collaborators for review and approval.

**Recommended composition of the CT:**

- Clinical researcher with experience in infectious disease research;
- Researcher with experience in systematic evidence review methodologies;
- Additional content experts as required depending on the nature of the outbreak;
- Administrative support.

**Responsibilities of the CT:**

- Alert the information specialist and systematic review team/s of the need for a RRNA.
- Review the protocol and identify if any adaptions or targeting are needed depending on the nature of the outbreak.
- Review the initial pilot search and systematic reviews identified to inform targeting of the final search (terms, restrictions in terms of sub-domain terms, and inclusion years), and protocol (targeting by disease, domains, sub-domains, level of study design to include, years to include, data charting and extraction scope).
- Share the protocol with collaborators and external partners to review as appropriate before starting the RRNA relay.
- Finalise and send the protocol together with a brief overview of the situation and clinical background information, to the search specialist and systematic review team/s by the end of Day 1.
- Provide a verbal briefing to the search specialist and systematic review team/s.
- Advise if specific language expertise might be required e.g. depending on disease endemic regions where evidence is likely to be generated.
- Register the protocol on the Open Science Framework (OSF) or similar platform.
- During the process the coordinating team will be at hand to answer clinical and content specific queries from the systematic review teams.

### Information specialist

The information specialist will be responsible for carrying out a systematic evidence search across all domain questions after the CT has reviewed and made any necessary amendments to the protocol depending on each disease. The information specialist will liaise with the CT and will carry out a pilot search for SRs and primary research to inform the final search. They will forward the search results, after de-duplication of records as an EndNote library to the CT ( Table 2). The CT will run an automatic full text article retrieval and upload the library with full texts retrieved to the systematic review program.

**Table 2.** Information specialist responsibilities.

**Day 1**	Provide specialist advice to refine the search terms and pilot the search to inform the final search strategy.
	Carry out an initial search for systematic reviews and a pilot search for primary research studies to inform targeting of the search strategy, protocol and resource planning.
**Day 1–2**	Carry out the systematic search of three databases using the final agreed search strategy, across the relevant domains when they receive the final protocol from the coordinating team.
	De-duplicate search records.
	Share the search results (i.e. an EndNote file), methods statement and search strategies with the SG and CT. The coordinating team sends out an alert when the results are uploaded to the systematic review software and ready to screen.
**Day 3–14**	On stand-by to support queries, evaluation of the search strategy as and if required. Provide information on the search strategy to inform the final report and publications.

### Systematic review team

To ensure rigour and quality, it is essential that the systematic review team/s involved are trained in the methodology and in screening and data extraction in advance. A minimum of three systematic reviewers is essential for quality. Depending on the volume of records retrieved, the number of systematic reviewers can be scaled up if required. The responsibilities of the systematic review team/s are outlined in Table 3, and ideal multidisciplinary expertise of the review team in Table 5.

**Table 3.** Responsibilities of the systematic review team.

**Day 1–2**	Review the RRNA protocol, set up the extraction templates and tools.
	Inform refinement of the protocol. Program the systematic review software; screening and data extraction templates. Identify additional reviewers with specific language skills if needed and feasible.
	Review the background briefings (e.g. from WHO/CDC websites) to familiar themselves with the disease.
	Establish an instant messenger chat platform (e.g. Slack) for all involved in the RRNA.
	Once the deduplicated EndNote search results are received, upload it to the systematic review software, run an automatic full text article retrieval, send an alert to all reviewers via the chat and e-mail to start screening.
**Day 2–6**	Screen titles and abstracts, followed by full text screening with data mapping.
	If full texts are not retrieved automatically, search for them in PubMed, Google, or the University online library. If still not accessible, exclude as inaccessible, document exclusion reason, and provide bibliography to these in the appendix of the final report.
	At full text screening stage, map key data including study design, domain, sub-domain and key population groups included (young children (<5 yrs), children & young people (CYP 5–17 yrs, adults (18+), pregnant women to provide a rapid overview of the available studies and inform data extraction prioritisation.
**Day 3–6**	When full text screening and data mapping is complete, review the interim data and liaise with the CT to discuss need for data extraction prioritisation by domain/sub-domain and study design ( Table 4). E.g. if sufficient high level of data is available to address a sub-domain, then lower level of evidence does not need to be extracted as the focus is to identify gaps in the evidence base.
	Start full data extraction of those domains and records pivoted to full extraction.
**Day 4–10**	Complete data extraction, clean the data – check for missing and inconsistent data for key data on: study setting study design, key focus and population groups, follow up as/if required to correct.
	Submit the data extraction tables for all domains to the coordinating team for a final review and data analysis. Analysis can be facilitated in advance by programming codes for descriptive analysis/tabulation and creation of graphs (Appendix 2–4), this will facilitate data dissemination and updates if required as new evidence emerges.

**Table 4.** Study designs and the hierarchy of evidence.

Hierarchy	Study designs
1a	Systematic reviews with meta-analyses
1b	Systematic reviews (without meta-analysis)
**Interventional studies**	
2a	Randomised controlled trials (RCT) (e.g., randomised treatment, supportive care, vaccine trials with control arm, Phase 2 and 3 vaccine* trials, adaptive RCTs
2b	Randomised controlled studies (e.g., randomised qualitative intervention with control arm)
	Non-randomised interventional studies (e.g. non randomised trials, before-and-after study /pre-post study/single arm, quasi-experiment, Phase 1 and non-randomised Phase 2 vaccine studies)
**Observational studies**	
4	Cohort studies* (prospective/retrospective) *a group of individuals exposed to a putative risk factor with or without an unexposed control group followed over time to determine the occurrence of disease. Participants selected based on the exposure status.
5	Case-control studies* e.g., cases of people and matched controls, human samples and control samples, diagnostic development studies with control *compares two groups of people or human samples: those with the disease or condition under study (cases) and a group of people who do not have the disease or condition (controls). Participants selected based on the outcome.
6	Cross-sectional studies (e.g. survey/focus groups, healthcare data analysis, diagnostic studies without control, outbreak reports) * measures the prevalence of health outcomes or determinants of health, or both, in a population at a point in time or over a short period. Participants selected based on the study inclusion and exclusion criteria.)
7	Case series/ Case reports
8	Opinion pieces (e.g. comments, letters, viewpoints) if they present primary data (for new and emerging viruses, early data may be presented in rapid comments)

**Table 5.** Systematic review expertise.

Minimum of three experienced systematic reviewers trained in the methodology, familiar with infectious disease research, prevention and response, the systematic review software and tools used for the RRNA.
Ideally based in different time zones to allow scalability and to bring in diverse language skills and expertise, e.g. knowledge of diseases endemic in different regions.

The review teams will be sent the protocol, with any edits made visible in track changes on Day 1. The team will refine the systematic review software if required.

The lead systematic reviewer will upload the EndNote file to the systematic review software. The reviewers will then start the screening (titles and abstracts, followed by full text data mapping and extractions), and will extract the data into a pre-defined data extraction template using the review software or alternatively directly in Microsoft Excel.

The systematic review team/s will export and send the completed data outcome templates to the coordinating team by mid-day by day 10. The systematic review software enables screening and data extraction processes to run in parallel and reduce the need for lengthy handovers, while minimizing knowledge transfer loss.

### Systematic review software

We will use systematic review software to allow processes to run in parallel without the need for handovers, standardisation of extractions, mapping of data and future updates as new evidence emerges. The systematic review software also enables continuous monitoring of progress, rapid identification of issues and facilitate audits and quality checks.

### Reporting responsibilities

The systematic review team/s are responsible for submitting the completed data extraction tables to the coordinating team via e-mail. The reviewers will complete and submit the PRISMA diagram, presenting the number of records identified from different sources, reasons for exclusions and final numbers included. This will be presented in the final report.

### RRNA process

The process is derived from modified systematic and rapid evidence review methodologies ( Figure 3).

**Figure 3.**
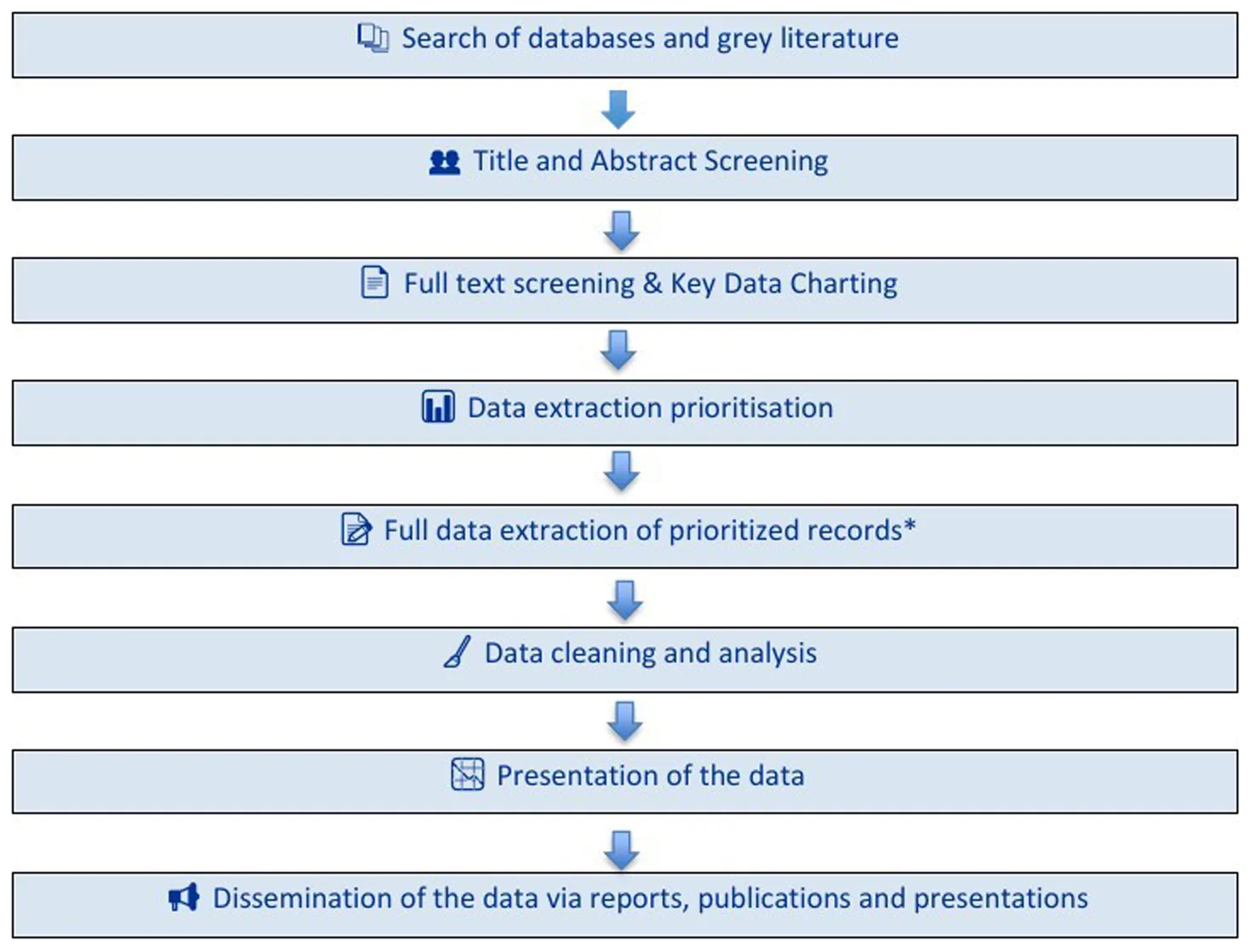
RRNA process flowchart

### Research domain categories

We will leverage our protocol to create an in-depth, ‘living’ RRNA platform presenting evidence across key health protection domains for different population groups. We will focus on specific priority pathogens/diseases or pathogen families. This framework will support continuous, needs-driven identification of evidence available across at-risk population groups for topics listed below:

1.  Clinical characteristics, epidemiology

2.  Immune response, seroprevalence

3.  Transmission (human-to-human) *

4.  Risk factors for infection, severe disease

5.  Vaccine and therapeutic prophylaxis research

6.  Diagnostic methods

7.  Therapeutic research

8.  Supportive care

9.  Social and behavioural factors

*A research domain focused on animal-to-human transmission is presented in the appendix as an add-on option (Appendix 1).

Evidence will be identified through a peer-reviewed search, complemented by grey literature (international, regional/national public health, WHO, CDC databases) search when required. We will search for studies including humans or human samples. No language restriction. By utilising a global relay of trained reviewers and a systematic review software ensure effectiveness, quality and minimise knowledge transfer loss.

We will perform a descriptive analysis and present interim analysis to stakeholders as appropriate and incorporate feedback received before finalising the report. We will disseminate the data directly to policy makers and via our Pandemic PACT website, reports and publications as appropriate to facilitate wide dissemination of the findings.

### Eligibility criteria

Studies focused on new and (re-) emerging priority infectious diseases of epidemic and pandemic potential with a limited evidence base, focused on at least one of the domain topics in the protocol and includes humans or human samples ( Table 6).

**Table 6.** Eligibility criteria.

Inclusion criteria	Exclusion criteria *
Studies focused on the specified disease/s that involves humans or human samples.	Pure animal, vector (insect, parasite), virus/pathogen studies, Data modelling studies.
Studies presenting primary data and focused on at least one of the research sub-domains in our protocol ( Table 7).	Pure cell culture and *in vitro* studies.
Any language.	Conference abstracts, Book chapters, Narrative reviews, Scoping reviews, Mapping reviews, Opinion pieces not presenting primary data, Pre-prints *

*These will be reviewed and modified depending on each outbreak and context. E.g. for new, emerging diseases with emerging evidence expected, pre-prints may be included, for studies that has been detected years back, systematic reviews may be relevant to include

**Inclusions:**

Studies focused on the disease;

     AND

•   Includes human or human samples e.g. (blood, semen, urine, faeces)

     AND

•   Focused on studying at least one of the RRNA research sub-domain categories in Table 7

•   No language restriction

**Table 7.** Data extraction table. Grey text indicates data fields that can be added for extracting additional information, depending on need and resources.

Step 1: Key Data Charting	
Demographics	For all studies extract:
**Bibliography**	Authors, Year, Title, Journal, Doi/weblink (if grey lit. website include date accessed)
**Disease/s**	Multiple choice if including more than one disease (e.g. viral family RRNA)
**Domains and Sub-domains**	Multiple choice x =1 – 9, If x, populate and select sub-domains for x – tick all
**Participants**	Number
**Key population groups**	Neonates/Infants (0–<1 yrs), Young Children (1 – <5 yrs), Children & Young People (CYP) (5–17yrs), Adults (18+), Pregnant women, NR
**Cases**	Number
**Cases: key population groups**	Neonates/Infants (0-<1 yrs), Young Children (1–<5), CYP (5–17), Adults (18+), Pregnant women, NR
**Study type**	Interventional, Observational
**Control group included (Yes/No)**	Yes, No
**Study design**	SR (systematic review), RCT, Randomised study, Non-randomised study, Cohort study, Case-control study, Cross-sectional study, Case series/report >10 cases, Case series/report ≤10 cases, Opinion piece with primary data, Other (specify):
**Study Year/s**	YYYY – YYYY or NR
**Study Setting (Country)**	Multiple choice
**Study Abstract**	Copy in (if not abstract is available, copy in the main summary of the study)
Step 2: Records prioritised for full data extraction	
Participant characteristics:	
**Age (mean/median/range):**	-Minimum age (youngest person included in study) -Maximum age (oldest person included in the study) -Median/Mean (IQR/ SD/ range) as presented or NR
**Sex**	Females include – Y/N/NR, If yes: (% , n/N) females included, NR
**Ethnicity**	Arab, Asian, Black, Indigenous (specify), Mixed, White, Other ethnicity (specify): , NR
**Occupational risk populations**:	Emergency Responders, Farmers/Herders, Funeral staff, Healthcare workers, Military workers, Other animal workers, Slaughter staff, Veterinarians, Waste workers/Cleaners, Wildlife/Forestry workers/Hunters, Other (specify), NR
**Specific population groups:**	Breastfeeding women, Care home residents, Drug users, General Population Healthcare workers, Hospital patients, Malnourished, Men who have sex with men, Other sexual/gender minority groups, Obese, People living with disabilities, People living with HIV, People living with other comorbidities (specify:), Prisoners, Pregnant women, Refugees / Internally displaced persons / Migrants, Sex workers, Students, Other specified vulnerable population groups (specify:), NR
**Healthy participants/samples**	Number
**Case contacts (n)**	Number
Domain	Sub-domain questions
**1.Clinical characteristics, epidemiology** **Sub-domains:** Signs & Symptoms Case Fatality Rate (CFR) Biomarkers (symptoms, lab, genomic/proteomic) Complications: acute Complications: pregnancy Complications: long term	• Cases (confirmed and probable) (n) • Case fatality rate (%, n/N) • Signs and symptoms (dropdown list – see Excel file) • Key clinical features that may distinguish the disease from other diagnoses studied? Yes No, If yes, extract the data: • Acute complications reported (e.g. liver, kidney, respiratory failure, secondary bacterial infection, sepsis, psychological etc.) – if yes, specify data: • Pregnancy complications reported Yes, No, NR • Long-term sequelae or complications (post-acute) studied, if yes, specify: • Biomarkers for diagnosing / identifying the disease studied? Yes No, if yes – tick box: a laboratory marker (e.g. CRP), clinical sign (e.g. hypertension), OR genomics, OR proteomics, OR a model (e.g. a clinical score system) OR a clinical validation system or other biomarker (specify): • Biomarkers/indicators for a risk of severe disease studied? Yes No, if yes – tick box: a laboratory marker (e.g. CRP), clinical sign (e.g. hypertension), OR genomics, OR proteomics, OR a model (e.g. a clinical score system) OR a clinical validation system (several established clinical markers, e.g. CRP, procalcitonin, blood pressure, and fever, etc.), or other biomarker (specify): • Data on risk of re-infection after natural infection? Yes No
**2. Immune response/Seroprevalence** **Sub-domains:** Acute immune response Seroprevalence Re-infection after infection	• Data on: dropdown: T cells, CD4+ / CD8+ cytokines, other? • Seroprevalence data? Yes No • Data on risk of re-infection after natural infection? Yes No
**3a. Transmission (human-to-human)** **Sub-domains:** Pathogen in human samples Transmission routes (human-to-human) Transmission risk/infectivity from human samples Effectiveness of healthcare IPC Effectiveness of PPE	• Incubation period? Yes No NR • Asymptomatic, prodromal, post-acute transmission reported? Yes No NR • Routes of human-to-human transmission studied (e.g.. airborne, direct contact, droplet, indirect contact, saliva, sexual)? • If yes, extract the data: • Virus detected in human samples? If yes, indicate sample type: • Risk of infection from human body fluids studied? ⚬ (if yes, indicate: blood, conjunctival fluids, breast milk, saliva, semen, stool, transplant tissue, urine)? ⚬ If yes, period/length of infection /persistence of virulence/virus in body fluids – extract the data. • Effectiveness of infection prevention control methods to reduce risk of transmission in healthcare settings? Yes No • Effectiveness of personal protective equipment (e.g., facemasks, eye shields) studied? Yes No
**4. Risk factors for infection and severe disease** **Sub-domains:** Risk factors for disease Risk factors for severe disease	• Host risk factors for infection identified (e.g. sex, age), comorbidities)? if yes, specify: • Host risk factors for severe disease or acute complications studied? if yes specify: • Host risk factors for long-term complications/sequela identified? If yes, specify:
**5. Vaccine & therapeutic prophylaxis** **Sub-domains:** Vaccine studies Vaccine antibody response Post-exposure prophylaxis	A. **Human vaccine tested?** If yes extract data on: • Name of vaccine: • What stage is it tested at?(dropdown list: Phase 1, Phase 2, Phase 3, Phase 4, Unknown) • Which populations is it tested in? tick all Neonates/Infants (<1 yrs), Young children (1-5yrs), CYP (5-17 years), adults (18+ years), pregnant women, any other specific population (specify), NR • Specify the number of doses: (drop-down options: 1, 2, > 2 , unknown) -If > 1 dose, specify the dose interval: (specify months; years, NR) • Route of vaccine: oral, intranasal, injected (subcutaneous, intramuscular), other • Data on the effectiveness of the vaccine in preventing? (select): Disease , Severe disease, Transmission, Re-infection? • Data on vaccine effectiveness against different virus strains? Yes, No • Data on vaccine effectiveness against different pathogens? Yes, No, If yes, which ones? • Data on waning antibody response after vaccination over time? Yes No • Data on risk of re-infection after vaccination? Yes No: B. **Therapeutic prophylaxis studied pre- or post- exposure prophylaxis)**? Yes No, If yes, extract data on: • Pre-exposure, Post-exposure, NR (tick all) • Name of Therapeutic: • Populations tested on: Neonates/Infants (<1 yrs), Young children (1-5yrs), CYP (5-17 years), adults (18+ years), pregnant women, any other specific population (specify), NR • Data on effectiveness: Yes No NR
**6. Diagnostics** **Sub-domains:** Rapid Diagnostic Tests (RDT) Other diagnostic tests	• Diagnostic tests trialled/tested/evaluated? Yes No • If yes, specify type of test: (Dropdown menu: ELISA, immunofluorescence, nucleic acid test, Rapid Diagnostic test (RDT), RT PCR, Sequencing Serology, Viral culture, other: specify or NR)? • Which samples were used/tested? (Dropdown menu: Blood, Cerebrospinal fluid, Dried Blood Spots, Faeces, Nasopharyngeal swab, Sputum, Tissues (biopsies/post-mortem), Urine, Other (specify): or NR) • Is it a multiplex assay? Yes No • If yes which viruses can the test detect? Free text or NR
**7. Therapeutics** Therapeutic studies	• Therapeutic treatment studied? Yes No, If yes, • Name of therapeutic/s studied : • Specify administration route/s for each tested (e.g. oral, I.V.) or NR: • Populations included in the study , tick all: Young children (5yrs), CYP (5-17 years), adults (18+ years), pregnant women, any other specific population (specify), NR • Data on effect of therapeutics on (tick box answers): (risk of transmission; Reduction in isolation period; recovery time; length of hospital stay; risk of severe disease; fatality rate, other: specify) studied? • Any severe* side effects identified? Yes No
**8. Supportive care** Supportive care studies	• Effectiveness of supportive care (e.g. oxygen, fluids, pain relief, psychological) studied? Yes No • If yes, what supportive care was studied? • Data on effectiveness? Yes, No, NR • Any severe* side effects identified? Yes No
**9. Social and behavioural factors** **Sub-domains:** Knowledge Attitude Access Uptake Healthcare implementation	This includes studies focused on evaluating the below and those focused on exploring factors that impact on the below (e.g., stigma, trust, engagement, information, socioeconomic etc.) • **Knowledge of** (dropdown list: Diagnostics, Disease, PPE, Prevention or control methods, Prophylaxis, Risk factors for transmission/ infection, Severe disease, Treatment, Vaccines, Other (specify:)). • **Attitude to** (dropdown list: Disease, Medical countermeasures (diagnostics, treatments, vaccines), Risk factors for transmission/infection/severe disease, Prevention or Control methods, Other (specify:)). • **Access to** (dropdown list: Diagnostics, Information/guidance, post-exposure prophylaxis, PPE, Prevention and control tools, Therapeutics, Supportive care, Vaccines, Other (specify:)) • **Uptake of (**dropdown list: Diagnostics/guided or self-testing, Post-exposure prophylaxis, PPE, Prevention and control methods, Therapeutics, Vaccines, Other (specify:)) • **Are there studies exploring healthcare staff** (dropdown list: Training in identifying or managing the disease, Capacity/capability in managing the disease, Facilitators/challenges to implementing care/IPC, Other (specify:)

**Abbreviations:** CYP: Children & Young People, IPC: Infection Prevention Control, NA: Not applicable, NR: Not reported, PPE: Personal Protective Equipment

**Exclusions:**

•   Studies not focused on the disease/pathogen

•   Studies focused only on animals, vectors (insects, ticks), virus, data modelling

•   Conference abstracts, book chapters, non-systematic reviews (scoping reviews, narrative reviews, mapping reviews)

•   Opinion pieces unless they present primary data (during outbreaks rapid early data may be published in a rapid letter/commentary)

Depending on each disease, systematic reviews and pre-prints may be included or excluded.

E.g. for diseases that were detected decades ago, it may be of relevance to include systematic reviews. For new diseases and ongoing outbreaks, it may be important to include pre-prints, as they are a means to rapidly disseminate data during ongoing outbreaks. However, for well known viruses older pre-prints are likely to be duplicated in an updated peer-reviewed publication, and therefore pre-prints may be excluded. This decision will be made for each RRNA, and will be communicated in the protocol, programmed in the review software to guide the reviewers, and communicated to the reviewers.

### Evidence search strategy

The databases to be searched will be reviewed and modified depending on the nature of the outbreak by the coordinating team. An information specialist will be responsible for searching three databases. They will pilot the search strategy for each RRNA, and after presenting the pilot search and consultation with the CT, finalise the search strategy and conduct the final search. As the focus is on identifying gaps in the evidence base and rapidly during the outbreak, it is important to ensure the search terms are inclusive but also specific when needed to reduce noise.

There are two main search strategies that will be considered:

- Search of all databases using the name/s for the disease and pathogen may be sufficient, to cast a wide net when the evidence base is limited. If a disease is set in a non-English speaking region, we will do an initial search for names of the disease in the languages spoken in this region, and if needed will add these to the search.
- Search all bibliographic databases of peer-reviewed literature using combinations of search terms presented below. For targeted RRNAs focused only on a selection of domains, these will be edited as appropriate.
- These searches will be complemented by a manual search of grey literature by the CT and review team using search terms based on the disease and pathogen name, to include primary data presented in grey literature, e.g. in outbreak reports ( Table 8).

**Table 8.** Databases to be searched for primary research.

MEDLINE (PubMed), Ovid Embase, Scopus, Cochrane Systematic reviews *, Epistemonikos *
Grey literature ^: Google, WHO, Regional CDCs, Regional/National Public Health, Ministry of Health websites in endemic countries and or countries of ongoing outbreak/s.

*Exclude if only focusing on primary research e.g. for newly emerging diseases ^To be adapted for each disease and context as relevant.

**Search timeframe:** To be defined depending on each disease and need, e.g. from database inception or from date of disease detection in humans, or from first large outbreak when evidence may be expected to be generated. For rare diseases we will search from inception, for diseases where the pilot search identifies a large amount of records it may be modified to a relevant more recent year to include up to date evidence and recent systematic reviews that will likely cover the earlier studies, for relevance and feasibility.

**For updates:** From the final inclusion date of the previous RRNA

**Search terms:** The search strategies use keywords and thesaurus headings, where available, to search in text words and controlled vocabulary, in conjunction with terms relating to the disease AND the research domains and sub-domains AND humans (human*, patient*, participant*, infant*, child*, female, male, pregna* , case*, woman, women, man, men, child, neonat*). The terms for the different questions (domains and sub-domains) are: incubation, symptom*, severity, “natural history”, “clinical features”, transmission, infectiousness, vaccin*, prophyla*, chemoprophyla*, prevention, protection, diagnosis, diagnostic, RDT*, screening, detection, immunity, immune*, antibod*, serology, waning, treatment, management, therapy, drug*, intervention*,“supportive care”, fluid*, electrolyte*, supplement*, mortality, death, “adverse events”, “side effect*”, complications, sequela*, immunocompromise*, “personal protective equipment”, “infection prevention control”, knowledge, attitude*, behavio*, practice*, social, acceptance, uptake, adherence, trust, stigma, “engagement”, communication, ethical, implementation, access, guidance, protocol, staff, training, management, capacity, capability, effectiveness

Exclude: In the search, where possible, book chapters, conference abstracts and references with "scoping review", "narrative review", "mapping review", or “systematic review” (if excluded) in the title field, with an animal and vector exclusion filter if relevant and if the team agree to use one. References retrieved will be exported to EndNote 21 (Thomson Reuters, New York, NY), and will be deduplicated automatically using EndNote, followed by manual deduplication to identify those not already retrieved by the software.

### Grey literature search strategy

The peer-review search may be complemented by a grey literature searching Google, WHO, Regional CDCs, National Public Health and Minister of Health websites. This is especially important for new and rare diseases where the evidence base is expected to be limited, or for diseases set in non-English speaking countries where data may not always be published in English journals. The grey literature search will be carried out manually by the CT and reviewers by defining search terms to use for each website, based on: ‘disease name’ AND ‘human*’. Note: if the disease is mainly known by a non-English name in the endemic countries, searches for both the English and the local disease name, will be carried out searches for grey literature in endemic countries’ native languages when appropriate and feasible. For the search using Google, the date of the search will be recorded, and the records identified copied into an offline source e.g. Word, to facilitate the screening of the first 50 hits by title and abstract by two reviewers. The records included for full text screening will be manually uploaded into the systematic review software for full text screening, data charting and extraction.

### Retrieving full text articles

Full text articles will be retrieved through EndNote’s automatic functionality, supplemented by manually searching for articles using Oxford University library access, PubMed and Google since EndNote’s automatic functionality does not identify everything. If the full text article is not identified after searching these databases, then it will be excluded due to being non-accessible. Articles retrieved manually will be uploaded into the systematic review software. Articles can either be uploaded directly or saved on a computer for later bulk upload. For bulk upload, the articles may need to be renamed and saved with the systematic review software ID system before bulk upload. The articles that are eligible for inclusion but de-prioritised for data extraction can be listed in the appendix of the final report (Appendix 5).

### Screening

For newly emerging diseases where the evidence base is expected to be limited, it is key to ensure that the screening strategy is rigorous as well as inclusive. Therefore, title and abstracts and full text records will be screened by two reviewers independently. Any disagreements will be resolved through consensus or by a third reviewer. We may use an accelerated screening approach whereby one reviewer is needed for inclusion and two for exclusion. By using a systematic review software, this means that once the records (search results) are uploaded, work can be ongoing in parallel, as it facilitates keeping track of tasks that has been done and those left to complete without need for lengthy handovers.

For records in languages other than English, abstracts and articles will be translated using Google Translate before screening. Both the original article and the translated version will be uploaded into the systematic review software. If required, a reviewer with specific language skills will be consulted to support the review. For each RRNA, if key evidence is expected to be published in non-English languages, engaging reviewers or review teams from that region is recommended. If it is not possible to translate an article, a note should be made in the report to this effect. Studies not extracted, can be listed with full bibliography in the supplemental file, with the reason documented.

### Data extraction

The aim of the RRNA is to identify gaps in knowledge and the evidence base. This means that minimal data such as: Disease, Research Domains and Sub-domains, Study design, Key Population Groups (Young children (<5 yrs), Children & Young People (5 – 17 yrs), Adults (18+ yrs) and Pregnant women and Study Setting will be collected to identify evidence gaps across these key parameters. If data is available, ethnicity will be recorded ( Table 7). This data will be used to inform the need for full data extraction for all or specific domains and sub-domain research categories. For those diseases and domains where full data extraction is needed, these records will continue to full extraction, extracting more detailed Population parameters, Intervention (Exposure), Comparator, Outcome (PI(E)COs)) to identify gaps and provide an overview of existing evidence to inform research prioritisation and coordination. Data will be extracted into a pre-defined outcomes table.

***Step 1: Key data charting***

Key data will be extracted (chartered) into the pre-defined data template by one experienced systematic reviewer.

- A second reviewer will check up to 70% of the data.
- The final data will be exported into Excel for interim analysis.
- The data will be reviewed by the CT and reviewers to identify need for pivoting for further extraction across all or specific domains.
- If high level of evidence exists that addresses sub-domains, then data extraction can be prioritised so that lower level of evidence is not extracted. The bibliography of the non-extracted records may instead be listed in the appendix.
- The data prioritisation step will be informed by the initial key data charting analysis. Extraction may be prioritised so that if there are a high number of studies addressing a domain, extraction will be prioritised by study design, so that studies of higher evidence level ( Table 4) will be extracted.

***Step 2: Full data extraction***

- For those domains and sub-domains identified as requiring full data extraction, a decision regarding which level of study design to be extracted will be documented.
- Data will be extracted by one reviewer, with a second reviewer checking up to 70% of the data.
- Data reporting should include description of the populations covered with the numbers and proportions (% (n/N)) for each specific population group presented.
- Missing data should be recorded as: NR (not reported). Data that are not applicable to a study as Non applicable (NA).
- The result section should report a key summary of primary and secondary outcome data, and data covering study design, setting, n numbers and PI(E)COs: Population, Interventions (Exposure), Comparator, Outcomes.
- To facilitate data checking, cleaning and analysis, the main objectives and study abstract will be extracted. Further, data extracted concisely with sufficient context for another reviewer to interpret it. This includes ensuring that abbreviations are spelt out and checking that symbols, such as µ are presented correctly in the extraction sheet.

### Definitions

- **Cases**: Clinically or laboratory probable and confirmed cases as reported in the study
- **Previous cases:** Seropositive cases/samples
- **Contacts:** Contact of acute confirmed/probable case in case-contact studies
- **Healthy participants:** in case-control studies, participants in social and behavioural studies
- **Total participants:** All participants included in a study (e.g. cases + contacts)
- **Age ranges:** Neonates (<3 months), Infants (<12 months) Young Children (1 - < 5years); Children & Young People (CYP, 5–17 years), Adults (18+ years), Older adults (>65 years)
- **Ethnicity:** Arab, Asian, Black, White, Mixed, Indigenous (specify:), Other ethnicity (specify):

### * Vaccine study phases

- **Phase 1 Interventional, no control**. The vaccine is administered to a small number of healthy volunteers for the initial assessment of safety and immune response generation.
- **Phase 2: Interventional, usually with controls**. The vaccine is administered to a large number of volunteers from the target population groups, to further assess its safety and ability to generate an immune response. Usually includes a control group.
- **Phase 3: Interventional, controlled study**. Administered to thousands of volunteers and compared with a matched control group, receiving a comparator to assess vaccine effectiveness.
- **Phase 4: Post-approval study, observational or interventional:** To collect additional information about how well it works, and monitor safety among a larger number of participants and long term outcomes, safety surveillance, and to assess efficacy and in response to regulatory requirements.

### Data analysis and dissemination

The systematic review team/s are responsible for exporting the completed data outcomes tables from the review software and for collating, tidying, analysing and submitting the results in a Microsoft Excel file to the CT by day 10–12 of the RRNA process. During cleaning, key data to check for consistency include study design, study setting, population groups included, number of total participants and cases, and to check mapping to the correct domains and sub-domains. It is recommended to scan through each extraction row to check that these key data points are consistently extracted and add or update any errors or missing data spotted. The CT will do a second review of the data export for completeness and accuracy with a focus on these key parameters, and will coordinate or analyse the data descriptively. We will create analysis codes to facilitate analysis for future updates and new RRNAs, to facilitate rapid data dissemination for action. 

The results will be reviewed and signed off by the CT and SG, then disseminated as open access to inform research and public health prioritisation and coordination strategies. The CT will develop a generic final report template to facilitate timely dissemination of the data and future updates as new evidence emerges as require. We will not do risk of bias nor explorations of consistency, or meta-analysis since the focus is on identifying key gaps in the evidence-base across domains and population groups, and develop a rapid but systematic method for doing so both in preparedness time and during outbreaks as new evidence emerges. The strength of the studies will be assessed by study design, number of participants and cases included, for the purpose of identifying if there is gaps in the evidence base. We will provide descriptive analysis to present an evidence map and summary of the available evidence, we The data may be disseminated as a report or a dashboard via a dedicated website, directly to stakeholders, presentations and publications. We will disseminate the data with the bibliographies of the included study as open-access to facilitate dissemination, access and facilitate the ability for others to readily pivot to a full systematic reviews if needed.

If need arises, e.g. during an outbreak, the key data charted (mapped) in step 1 will be analysed rapidly to facilitate rapid identification of key gaps evidence across sub-domains by level of evidence and inclusion of different population groups (e.g. young children, CYP, adults, pregnant women), settings and study domains and if available data on ethnicity. This data can be disseminated rapidly to stakeholders, supplemented with links to the records, bibliographies and study abstracts to inform incident teams, research prioritisation and coordination during outbreaks.

### Quality control

Quality will be maintained by ensuring that the organisations and teams involved have previous necessary experience as described above and the resources needed. The protocol will be reviewed for each disease by experts in the infectious disease, domains included and by consultation with content experts as required and modified as appropriate. The search specialist and systematic reviewers will be engaged and trained in the protocol in advance. Ensuring that the teams have experience of systematic reviews will ensure rigour, consistency and quality. Using systematic review software will facilitate quality checks, audit and evaluation. By including a grey literature search and by engaging external experts with expertise in specific diseases, we will cross-check that key studies have been included in the peer-review search, and that the interpretation captures key needs. The final data identified will be reviewed by infectious disease specialist and by content experts with experience relevant to the specific outbreak and clinical domain questions before dissemination.

### RRNA updates

The RRNAs can be updated when new key evidence emerges, e.g. during and after outbreaks, or implementation of new therapeutic or vaccine trials. The decision to update the RRNA will be taken by the SG in collaboration with the CT and other stakeholders. Requests for an RRNA or update from external stakeholders will be reviewed by the SG to inform a decision for if there is need and resources to run the RRNA.

## Discussion

The outcome of the RRNA will be a summary of existing evidence across pre-defined research domains, by population groups and settings, to identify where there are gaps in evidence. This will provide baseline data to inform research prioritisation and coordination strategies in preparedness time and during outbreaks. As a secondary objective, the methodology facilitates pivoting to an in-depth evidence review, to inform clinical and public health strategies if required. 

This protocol is developed to facilitate rapid, systematic, robust appraisal of published evidence to identify gaps in the evidence base, to inform research prioritisation and coordination of research responses in preparedness time and during outbreaks. It is developed to facilitate implementation in any resourced setting and collaborative partnership working to optimise resources. We welcome others to adopt and adapt the protocol. We will establish and disseminate a baseline of RRNAs for high priority diseases, to provide a baseline to inform preparedness activities and for rapid update as new evidence emerges, with the aim to strengthen preparedness to new and (re-) emerging epidemic and pandemics to protect populations and improve outcomes.

## Data Availability

All data generated through the Living Rapid Research Needs Appraisal (RRNA) platform are curated and managed within the University of Oxford’s secure REDCap environment, hosted by the Nuffield Department of Medicine. The resulting datasets are made openly available via the **RRNA Project Figshare**, hosted under the **Pandemic PACT Figshare group repository** within the University of Oxford Figshare instance : https://portal.sds.ox.ac.uk/pandemicpact ^6^ Select visualisations of RRNA outputs will be available on the **Pandemic PACT public website** ( https://www.pandemicpact.org/rapid-research-needs-appraisals) ^7^ under the dedicated RRNA section. All underlying data and figures displayed on this platform can be downloaded directly or accessed through the linked Figshare repository. All code developed for data processing and visualisation is available under an MIT open-source license on GitHub: https://github.com/enovatedesign/pandemic-pact ^8^. Extended materials supporting the study - including the data extraction tables, evidence maps, bibliographies of eligible studies, and study characteristic tables; will be available via the RRNA Project Figshare, hosted under the Pandemic PACT Figshare group repository: ( DOI 10.25446/oxford.30480455) ^9^ and the **Open Science Framework (OSF)** repository: DOI 10.17605/OSF.IO/TWFNR ^10^. This project contains the following extended data: Map 1: Study settings Table 1: Evidence map Figure 1: Domains and subdomains by study design Figure 2: Domains/subdomains by population groups Figure 3: Medical countermeasures Table 1: Data extraction♠c Table 2. Study Characteristics Table 3: Bibliographies of eligible studies All extended data are open access under the Creative Commons Attribution 4.0 International License (CC BY 4.0). You may use, distribute, and reproduce this material in any medium, provided the original source and authors are credited. **Protocol registration**: https://osf.io/twfnr/ DOI 10.17605/OSF.IO/TWFNR

## References

[1] Ganann R Ciliska D Thomas H : Expediting systematic reviews: methods and implications of rapid reviews. *Implement Sci.* 2010;5: 56. 20642853 10.1186/1748-5908-5-56 PMC2914085

[2] Polisena J Garritty C Umscheid CA : Rapid review summit: an overview and initiation of a research agenda. *Syst Rev.* 2015;4(1): 111. 26407674 10.1186/s13643-015-0111-6 PMC4583747

[3] Antonio ES Alobo M Norton A : Lessons learnt from developing and applying research priorities during the COVID- 19 pandemic: reflections from the global research collaboration for infectious disease preparedness (GloPID-R). *BMJ Glob Health.* 2024;9(7): e015278. 39079896 10.1136/bmjgh-2024-015278 PMC11293373

[4] The World Health Organization: Pathogens prioritization: a scientific framework for epidemic and pandemic research preparedness.WHO. July2024; accessed 10 Sept, 2025. Reference Source

[5] Sigfrid L Moore C Salam AP : A rapid research needs appraisal methodology to identify evidence gaps to inform clinical research priorities in response to outbreaks-results from the Lassa fever pilot. *BMC Med.* 2019;17(1): 107. 31185979 10.1186/s12916-019-1338-1 PMC6560772

[6] Pandemi PACT Research Program: Pandemic PACT tracking research funding and evidence for action.Pandemic Sciences Institute, University of Oxford, UK, March2024; accessed 24 Nov 2025. https://www.pandemicpact.org/about/our-data

[7] Pandemic PACT Research Program: Rapid research needs appraisals.Pandemic Sciences Institute, University of Oxford, UK, March2024; accessed 24 Nov. 2025. https://www.pandemicpact.org/rapid-research-needs-appraisals

[8] Pandemic PACT: Enovate Design/Pandemic PACT. *GitHub.* accessed 24 Nov 2025. https://github.com/enovatedesign/pandemic-pact

[9] Pandemic PACT Figshare group: Extended data living Rapid Research Needs Appraisal (RRNA) for priority disease protocol. accessed 24 Nov. 2025. 10.25446/oxford.30480455

[10] Sigfrid L : A 'living' rapid research needs appraisal for priority diseases platform. *Open Sciences Framework.* 2023; accessed 24 Nov. 2025. https://osf.io/twfnr/overview

