# Supplementary material for: Living Rapid Research Needs Appraisal (RRNA) for Priority Diseases Protocol
Source: Wellcome Open Res. 2026 May 25;10:668. Originally published 2025 Dec 10. [Version 2] doi: 10.12688/wellcomeopenres.25003.2 (PMC13639346; doi:10.12688/wellcomeopenres.25003.2)
Supplement: Peer review history

# Reviewer report: Reviewer response for version 2

**DOI:** 10.21956/wellcomeopenres.29201.r168136

**Published:** 2026-10-05

**Authors:** Bruno Pozzetto

**Affiliations:** 1 CHU de Saint-Etienne, Saint-Etienne, France

As specified at the beginning of the manuscript, “this document presents a methodology for establishing a platform of ‘living’ rapid research needs appraisal (RRNA). The aim is to rapidly and robustly identify gaps in evidence across pre-defined research domains for new and re-emerging diseases of epidemic and pandemic potential, where existing evidence and knowledge is limited, to inform research prioritisation and coordination in preparedness time and during outbreaks.” I agree with the two other reviewers about the ambition of this project. I read attentively the protocol proposed by the authors and I globally agree with the methodologies that are proposed and that are in accordance with those usually recommended for conducting a review on an emerging subject.

 However I am a bit puzzled by the amount of expert work that will be required to conduct this kind of prospective evaluation for a single subject and to actualize regularly the document according to the proposed deadlines, given the high number of “new and re-emerging diseases of epidemic and pandemic potential” listed in different reviews on this subject. I would be interested to know the numbers of persons necessary to conduct such an ambitious task and which Institution (the University of Oxford?) would be prospectively in charge of the governance of this project. Who will decide about the prioritization of the topics and of size of the projects? For instance, if the topic of viral hemorrhagic fevers is chosen, will it be limited to a single agent (or one species of a viral genus) or will it treat simultaneously for instance all the viruses involved in a same geographic area or sharing the same zoonotic reservoir? I think that different methodologies and different timescales will be necessary according to the size of the treated subjects. In this perspective, I wonder whether a single methodology is adapted. Do the authors tested their protocol on a few instances of recent (re-)emergencies (i.e; arboviral disease as Oropouche, zoonotic disease as *Orthohantavirus andesense*, or *Orthoebolavirus bundibugyoense*). Such experiments of the protocol would be useful to test its practical feasibility.

 Another question addresses the place of IA-tools in the generation of this kind of bibliographic inquiries. I saw that the other reviewers addressed the same question. If the project is hosted by the University of Oxford, I think it seems feasible to incorporate an IA-driven assistance to the projects so that it could be possible to restart automatically the protocol without needing to activate again all the circuits described in the flowchart of the project.

 I am sorry not to be more helpful for improving this ambitious task. Globally I adhere to the idea of conducting prospectively a bibliographic surveillance of infectious (re-)emergencies, even if I stay a bit puzzled by the means needed to conduct this surveillance at a large scale with human means based on traditional reviewing processes. Regarding the protocol, I endorse the general organization, even if the times scheduled for the first days in Figure 2 seem a bit under-estimated for all the tasks that are listed.

 All my best wishes with this helpful project for the community.

Is the study design appropriate for the research question?

Partly

Is the rationale for, and objectives of, the study clearly described?

Yes

Are sufficient details of the methods provided to allow replication by others?

Yes

Are the datasets clearly presented in a useable and accessible format?

Yes

Reviewer Expertise:

Medical virology - Public Health

I confirm that I have read this submission and believe that I have an appropriate level of expertise to confirm that it is of an acceptable scientific standard.

# Reviewer report: Reviewer response for version 1

**DOI:** 10.21956/wellcomeopenres.27542.r144794

**Published:** 2026-02-05

**Authors:** James Scott Parrott

**Affiliations:** 1 Rutgers University, Newark, New Jersey, USA

This is a very ambitious effort to create living rapid research needs appraisal (RRNA) pipeline and platform. If feasible, it should provide useful information for evidence synthesis professionals, clinicians and policy makers regarding infectious disease outbreaks.

 The process, as described, maps to standards for gap analysis or rapid reviews.

 As this is a protocol, I interpret the goals (specifically the timeframe) as aspirational rather than (at least currently) realistic. Whether they are able to (or how often or under what conditions they are able to) meet their targets as specified in Figure 2, I take to be an empirical question. As this is a data charting effort (rather than a systematic review where ROB and results extraction are expected), it may be realistic—though I have my doubts but would be thrilled to be proved wrong.

 I did have some questions about specific details:

 It wasn’t entirely clear to me what would count as a trigger or how this monitoring would be systematically carried out.

 There is very little information regarding the software/platform that will be used. Given that, on p.7, they make the statement, “will extract the data into a pre-defined data extraction template using the review software or alternatively directly in Microsoft Excel” leads me to believe that they have not yet worked out the basic specifications of the system that would be needed. The following statement is completely unrealistic regarding software/platform updates, “The review teams will be sent the protocol, with any edits made visible in track changes on Day 1. The team will refine the systematic review software if required.” I am wondering if they are considering (there does not seem to be a concrete plan) to adapt some of the open source evidence synthesis platform code like Eppi Reviewer or SRDR+ (now decommissioned but available on github). At any rate, all of this is left to the reader’s imagination. Without a highly integrated platform tailored for this specific use, I see the feasibility of the timeline as unrealistic.

 I was surprised to see nothing about AI-scaffolded title/abstract screening as this has now become the standard within the evidence synthesis field.

 Regarding data charting and extraction (pp.13-14), the authors say, “A second reviewer will check up to 70% of the data.” This is very vague and could mean anywhere from a negligible amount to the majority. And, there are no rules specified for determining how many studies should be evaluated by a second reviewer.

 In summary, this protocol, in general provides an overview of an ambitious but useful project. There are some crucial details left out, but it may be that those details need to be worked out in situ rather than in the abstract.

Is the study design appropriate for the research question?

Yes

Is the rationale for, and objectives of, the study clearly described?

Yes

Are sufficient details of the methods provided to allow replication by others?

No

Are the datasets clearly presented in a useable and accessible format?

Yes

Reviewer Expertise:

Evidence synthesis methodology with a specific focus on data extraction and warehousing platforms.

I confirm that I have read this submission and believe that I have an appropriate level of expertise to confirm that it is of an acceptable scientific standard.

# Response

**Published:** 2026-04-15

**Authors:** Louise Sigfrid

**Affiliations:** Pandemic Sciences Institute, University of Oxford, Oxford, England, UK

Thank you for your review of our protocol, please find a point-by-point response below.    This is a very ambitious effort to create living rapid research needs appraisal (RRNA) pipeline and platform. If feasible, it should provide useful information for evidence synthesis professionals, clinicians and policy makers regarding infectious disease outbreaks.

 The process, as described, maps to standards for gap analysis or rapid reviews.

 As this is a protocol, I interpret the goals (specifically the timeframe) as aspirational rather than (at least currently) realistic. Whether they are able to (or how often or under what conditions they are able to) meet their targets as specified in Figure 2, I take to be an empirical question. As this is a data charting effort (rather than a systematic review where ROB and results extraction are expected), it may be realistic—though I have my doubts but would be thrilled to be proved wrong.  

**Response: **We agree that the timeline presented in the protocol is particularly relevant to outbreaks, when evidence gaps need to be identified rapidly to inform research response prioritisation for effective use of limited resources. Our pilots have shown that it is feasible, particularly if a relay of reviewers collaboration across time-zones is available, supported by review software. The feasibility will depend on the volume of records available, which is why it is developed in a modular approach whereby it can be targeted as relevant and if needed, e.g. restricted to key domains and by search time-frame. By establishing the methodology we have strengthened preparedness for timely implementation, and also developed an online training program to facilitate scale-up. Once an RRNA has been implemented, future updates can be implemented very rapidly, e.g. during prolonged outbreak where there is a need to revisit prioritisation as new evidence emerges.

 I did have some questions about specific details:

 It wasn’t entirely clear to me what would count as a trigger or how this monitoring would be systematically carried out.

- **Response: **We have clarified further in the protocol that a trigger is an event such an outbreak or a public health emergency, and for updates, as new evidence is available which for most domains will be as new outbreak emerges.

There is very little information regarding the software/platform that will be used. Given that, on p.7, they make the statement, “will extract the data into a pre-defined data extraction template using the review software or alternatively directly in Microsoft Excel” leads me to believe that they have not yet worked out the basic specifications of the system that would be needed. The following statement is completely unrealistic regarding software/platform updates, “The review teams will be sent the protocol, with any edits made visible in track changes on Day 1. The team will refine the systematic review software if required.” I am wondering if they are considering (there does not seem to be a concrete plan) to adapt some of the open source evidence synthesis platform code like Eppi Reviewer or SRDR+ (now decommissioned but available on github). At any rate, all of this is left to the reader’s imagination. Without a highly integrated platform tailored for this specific use, I see the feasibility of the timeline as unrealistic.

- **Response: **Thank you for your comment. We have clarified in the protocol that the coordinating team is responsible for ensuring that the review software is pre-programmed to enable rapid implementation and rapid refinements as appropriate for each disease, need and context.
- Further, that using a review software guides and facilitates standardisation of the extractions by limiting need for open ended extractions, which facilitates completion and quality assurance. The programme can be rapidly targeted, e.g. hiding domains or sub-domains or adding specific questions to a domain, since the protocol is designed in an adaptable, modular approach.

I was surprised to see nothing about AI-scaffolded title/abstract screening as this has now become the standard within the evidence synthesis field.

- **Response: **We agree that AI-assisted title and abstract screening has a potential to improve efficiency, and this may be used if available in the review software. However, we have designed the protocol to facilitate implementation in any resourced settings, recognising that access to review software may be limited. Hence, we have not included this, and in our experiences when implementing the protocol, existing technology has limited benefit for the purpose of the RRNA, which covers a wide range of domain topics. Further for emerging infections existing evidence is generally limited to lower study designs, and often contain inconsistent or incomplete reporting. This creates challenges for reliable AI-based identification without prior calibration and validation. This is definitely an area to explore further for the future as AI methodologies may become more reliable to apply and open accessible.

Regarding data charting and extraction (pp.13-14), the authors say, “A second reviewer will check up to 70% of the data.” This is very vague and could mean anywhere from a negligible amount to the majority. And, there are no rules specified for determining how many studies should be evaluated by a second reviewer.

- **Response: **Thank you for highlighting this. We have further refined this on page 18: “A second reviewer will check a minimum of 30% and up to 70% of the data, depending of volume of records, need and resources.”

# Reviewer report: Reviewer response for version 1

**DOI:** 10.21956/wellcomeopenres.27542.r142649

**Published:** 2026-01-05

**Authors:** Christian Morgenstern

**Affiliations:** 1 MRC Centre for Global Infectious Disease Analysis & WHO Collaborating Centre for Infectious Disease Modelling, Jameel Institute, School of Public Health, Imperial College London, London, England, UK

Thank you for inviting me to review this paper, which addresses a critically important subject. Access to high-quality data on infectious diseases at the outset of an outbreak is crucial to enable timely interventions, whether pharmaceutical or non-pharmaceutical (NPIs). The authors make a substantial contribution by setting out a protocol for the rapid review of research needs to support early outbreak response.

 One overarching observation is that the study protocol implicitly assumes that, for each outbreak, this work would start from scratch with no prior systematic review which could be utilised. This assumption may have been correct before the COVID-19 pandemic. However, initiatives such as the WHO Global Repository for Epidemiological Parameters (grEPI, https://github.com/WHO-Collaboratory/collaboratory-epiparameter-community?tab=readme-ov-file) by the WHO Hub for Pandemic and Epidemic Intelligence, supported by Imperial College London (ICL), the London School of Hygiene and Tropical Medicine (LSHTM) and the Public Health Authority of Canada (PHAC), in collaboration with a wide range of other institutions, provide a basis for the WHO Priority Pathogens already. Systematic reviews on four priority pathogens have already been published ^1–3^, alongside a publicly available database and an R package (epireview: https://github.com/mrc-ide/epireview).  

 For re-emerging diseases, grEPI, alongside other complementary databases, could serve as a starting point for informing the research community about the latest available data and estimates. Focusing on how that rapid update looks in an outbreak situation is challenging, and it would be excellent to see the authors expand significantly on the RRNA update section on page 15 (version 1 of the paper). For new pathogens, the study protocol provided is excellent, though I have several more specific comments.  

 All page numbers refer to version 1 of the paper:

- *Page 5, composition of the CT: *Besides clinical researchers, the core team should also include infectious disease epidemiologists, who are a key consumer of this data.
- *Page 5, systematic review team:* The authors state that to ensure rigour and quality, the systematic review team needs to be trained in advance. I agree with this, but it implies that the team must be established before any outbreak or rapid response. It would be helpful to state this more explicitly in the study protocol.
- *Tables 1 and 3:* Why is EndNote, as a specific system, mentioned in the protocol, but for all other tasks, the authors talk generically about the review software? Would it be better to say reference manager?
- *Table 4:* It might be helpful to say meta-analysis and data synthesis, as some data might not lend themselves to meta-analysis but could be synthesised using other statistical techniques (e.g. Bayesian Synthetic Likelihood methods).
- *Page 7, first paragraph:* What do the authors mean by ‘the team will refine the systematic review software if required’? Given the very tight timelines, is that feasible? What type of changes do the authors have in mind, and what do they mean by software in this context?
- *Page 7, second paragraph:* Is extracting into Microsoft Excel a realistic option if a global team does this across time zones? Should this not be captured in a robust database?
- *Page 7, Systematic review software:* Why is the systematic review software included in the study protocol? It would be better to abstract any software components from the study protocol and include a general section outlining what this software should be able to do. Furthermore, this paragraph reads as if the authors intend to use this study protocol solely for themselves rather than provide a generic tool such as PRIMSA, which is a shame, as the main benefit for the wider community is in that.
-  
*Page 7, reporting responsibilities*: Is e-mail an appropriate system to coordinate data? Should this not be done via a database that keeps records of amendments/changes, or via a tool such as GitHub, which also provides change tracking via commits and allows for reviews?
- *Page 7, research domain categories*: Point 3 should be expanded to include a broader range of data, including the full range of epidemiological parameters and models. Restricting to just human-to-human seems extreme, particularly for vector-borne diseases such as Zika.
- *Page 8, research domain categories*: Point 4, the risk factor outcomes seem narrow. Why not include other risk factor outcomes, such as death, as well?
- *Page 8, research domain categories*: One category which is missing and would be beneficial to add is Genetics. Understanding evolutionary mutation rates, phylodynamics, and phylogeography is essential. The other missing category is non-pharmaceutical interventions.   
- *Page 8*: The authors say that results will be disseminated through the Pandemic PACT website. Why is that included in the study protocol?
- *Page 9, Table 6*: The inclusion criteria are limited to human-to-human transmission, which seems too restrictive, especially for vector-borne diseases (e.g., Zika, Dengue). Why are data modelling studies excluded, as they are critical to domain 3? Do the authors intend to exclude theoretical modelling studies that are not fitted to data? Having an asterisk on preprints is ambiguous, and it might be better to state that during non-outbreak periods, preprints are explicitly excluded, but during outbreaks, they are included. Also, why are systematic reviews not excluded? If data are extracted and a meta-analysis is performed, the analysis would consist of study results and summary estimates, potentially double-counting some studies but not others, leading to biased estimates. I understand that the aim is to use systematic reviews to map the existing evidence base. Still, the protocol needs to treat systematic reviews more carefully so that they are not included in any meta-analysis or data synthesis.
- *Page 9, Table 7*: Step 1: Cases should account for the possibility that some papers may report on multiple outbreaks. Study years would be better captured as study dates, allowing the extraction to capture actual dates if available. Step 1 is suitable for capturing the overall information for the paper; however, it is critical to capture the context for each estimate in each sub-domain, as these may vary (by age group, risk population, location, number of observations/cases, etc.) and could introduce errors in subsequent analysis, particularly if other groups use the extracted data for follow-on work, if not captured accurately.
- *Page 10, domain 1*: CFR is the case fatality ratio. This is not a rate because it lacks an associated time unit. If the authors prefer to call it a risk, that would be okay, but it is not a rate. Also, it should be captured if this CFR estimate is a naïve or adjusted estimate (or unspecified).
- *Page 10, domain 2*: Do the authors intend to extract the seroprevalence data? This would be of enormous benefit, but it requires much more information in the study protocol, as this is a complex task. Also, this would require the extraction team to be trained and to have sufficient knowledge of serology. This is another example in the manuscript where it is unclear whether the aim is only to highlight gaps or to actually extract data, which is a much more difficult task.
- *Page 10, domain 3*: Why is only the incubation period being extracted/recorded? This should extend to all delays, including the serial interval, the onset-to-hospitalisation interval, etc. Also, it would be great to capture whether only sample summary statistics (mean, median, min, max, etc.) are reported or whether distributions are fitted.
- *Page 10, domain 3: *It would be good to capture reproduction number estimates. More generally, several transmission parameters, such as overdispersion, should be included in this domain to enable infectious disease epidemiologists to build models.
- *Page 12, search terms*: Thank you for including the search terms. In an outbreak situation, this is only possible since the last RRNA update, as otherwise one would get too many titles and abstracts to screen (e.g., for Ebola or SARS-CoV-2, this would exceed 10,000 papers if the databases are searched since inception).
- *Page 13, Screening*: Has the use of Google Translate been tested and validated for inclusion in the study protocol? Is there a risk that the translation introduces errors?
- *Page 13*: A second reviewer will check up to 70% of the data – this is huge. Why can’t there be some double extraction at the beginning until the extractors converge to a consistent extraction? Otherwise, this may not be feasible given the time frame.  
- *Page 14, full data extraction*: This would be nice, but how feasible is that unless the team is already in place, fully trained, has all the tools at their disposal and only needs to update since the last entry into the database? Even in the most minimal form, this would be a considerable effort, especially if up to 70% of extracted data is double-checked.
- *Page 14, Definitions*: Please be more specific about the case definition.
- *Page 14, Definitions*: The age ranges should be more flexible, as each paper will use its own definition.
- *Page 14, data analysis and dissemination*: The authors state that they will provide the analysis code. Why is this included in a study protocol? Would it not be better to set out the requirements and standards for the analysis? Also, several tools already exist for this (epiverse, epireview) and are in use.
- *Page 14, data analysis and dissemination*: In the second paragraph, the authors provide a helpful discussion of what should be done during preparedness time versus during an outbreak. It may be helpful to consider existing work in this area ( https://mrc-ide.github.io/epireview/) as well as broader WHO initiatives, such as grEPI.
- *Page 15, RRNA updates*: It would be helpful if the authors could expand this section substantially. This is non-trivial, and setting this out in the protocol would be helpful. A good example is how database entries should be updated between preprints and the final published paper.

In the discussion, the authors raise the essential point of collaborative partnerships, which will be critical to this type of work. However, the key question is who convenes and funds this (not to be answered by this paper).

 During non-emergency periods, systematic reviews with meta-analysis and data synthesis should be strongly preferred as the gold standard. These reviews should be designed to be updated, and the underlying extracted data should be publicly available. This paper fills a critical gap in the literature on how these datasets can be rapidly updated to support outbreak response. Overall, this is an excellent contribution to the literature, but it would benefit from a more precise separation of the study protocol from tools such as the systematic review software, reference manager, or dissemination tools. For the study protocol itself, it would be helpful to separate the use case of mapping existing data from that of extracting it, which is a more complex task.

Is the study design appropriate for the research question?

Partly

Is the rationale for, and objectives of, the study clearly described?

Yes

Are sufficient details of the methods provided to allow replication by others?

Yes

Are the datasets clearly presented in a useable and accessible format?

Not applicable

Reviewer Expertise:

Infectious disease epidemiology

I confirm that I have read this submission and believe that I have an appropriate level of expertise to confirm that it is of an acceptable scientific standard, however I have significant reservations, as outlined above.

## References

[1] : Marburg virus disease outbreaks, mathematical models, and disease parameters: a systematic review. *The Lancet Infectious Diseases* .2024;24(5) : 10.1016/S1473-3099(23)00515-7e307-e317 10.1016/S1473-3099(23)00515-7 PMC761587338040006

[2] : Ebola virus disease mathematical models and epidemiological parameters: a systematic review. *The Lancet Infectious Diseases* .2024;24(12) : 10.1016/S1473-3099(24)00374-8e762-e773 10.1016/S1473-3099(24)00374-8 PMC761662039127058

[3] : Lassa fever outbreaks, mathematical models, and disease parameters: a systematic review and meta-analysis. *The Lancet Global Health* .2024;12(12) : 10.1016/S2214-109X(24)00379-6e1962-e1972 10.1016/S2214-109X(24)00379-6 39577970

# Response

**Published:** 2026-04-15

**Authors:** Louise Sigfrid

**Affiliations:** Pandemic Sciences Institute, University of Oxford, Oxford, England, UK

Thank you for your thorough review of our protocol, please find a point-by-point response below:

- We have now clarified the recommendation to search for existing systematic reviews to inform targeting of the protocol and expanded on the updates.
- See: Table on page 8: “To review the need for refinement of the protocol depending on the disease and context. Consult with experts and carry out an initial search for available systematic reviews already addressing any of the domains, and target the protocol accordingly.”
- AND On page 16: “The protocol and programmes are developed to facilitate regular updates as and if required as new evidence emerges. For emerging and re-emerging infectious diseases new evidence is most likely to be generated during or after outbreaks, therefore there is no fixed time-frame stipulated for updates. Updates searches are recommended to be done from the final inclusion date of the previous RRNA.”
- AND on Page 16, Table 6. We have deleted the Systematic review databases, which were incorrectly entered in this table.

 

- We have also clarified the aims and remits of the RRNA further in the background on page 4, which addresses a number of the questions below:

 

- “ The focus is on identifying gaps in the evidence base across domain topics and for different at risk populations and settings. As a secondary utility, if sufficient evidence is identified for a domain, it can readily be pivoted to a full systematic review if required and resources allows. The benefit of the RRNA is that it covers key domains relevant for prevention and early outbreak control to protect communities including healthcare workers This breadth of evidence would not be feasible to cover in a systematic review, however, if the RRNA identifies sufficient evidence for a specific domain it can readily be pivoted to a full systematic review if needed and resources allows. The RRNA methodology is specifically developed for new and (re-) emerging infectious diseases where existing evidence is limited. Diseases that have been well researched over the years, such as Dengue and COVID-19 are out of scope for a full RRNA, but could be considered for a very targeted RRNA focused on one domain or a few sub-domain questions.”

All page numbers refer to version 1 of the paper:

- *Page 5, composition of the CT: *Besides clinical researchers, the core team should also include infectious disease epidemiologists, who are a key consumer of this data.
- Thank you this has been updated – see page  “Additional content experts, such as infectious disease epidemiologists, public health professional, social scientist, diseases specific experts as required depending on the nature of the outbreak;”
- *Page 5, systematic review team:* The authors state that to ensure rigour and quality, the systematic review team needs to be trained in advance. I agree with this, but it implies that the team must be established before any outbreak or rapid response. It would be helpful to state this more explicitly in the study protocol.

 

- We have clarified the need to ensure there are staff pre-trained in the methodology on page 7 & 8: “It is recommended to sustain a  standing CT trained in the methodology to ensure readiness and rapid mobilization when needed. The CT will be responsible for coordinating the process from notification of the need for a RRNA from the SG, through to completion (Table 1). The CT will also be responsible for training reviewers in advance and for scaling up implementation during outbreaks. This protocol and associated training video can aid in scale up.”

 

- **CT responsibilities**: Signs-off the final protocol and any amendments. Ensures the protocol is registered in an open-accessible database such as the Open Science Framework and that the review software is pre-programmed, and all stakeholders involved are trained and familiar with the process and review software used.

- *Tables 1 and 3:* Why is EndNote, as a specific system, mentioned in the protocol, but for all other tasks, the authors talk generically about the review software? Would it be better to say reference manager?
- We have updated the text throughout to state “reference manager” rather than specifically EndNote.

 

- *Table 4:* It might be helpful to say meta-analysis and data synthesis, as some data might not lend themselves to meta-analysis but could be synthesised using other statistical techniques (e.g. Bayesian Synthetic Likelihood methods).
- Our screening is specifically set up to identify systematic reviews with meta-analysis, as these are clearly identifiable at the title and abstract screening stage. It would require additional resources to assess if other systematic reviews are using other analysis methods. The RRNA is developed to balance rigour with effectiveness to enable rapid response during outbreaks. Other systematic reviews will be captured amongst systematic reviews and can be pivoted to further assessments if required.

 

- *Page 7, first paragraph:* What do the authors mean by ‘the team will refine the systematic review software if required’? Given the very tight timelines, is that feasible? What type of changes do the authors have in mind, and what do they mean by software in this context?

 

- We have clarified that the program needs to be pre-programmed, which will enable rapid refinements as/if required on page 8:  “To coordinate the overall process and teams involved from the start through to dissemination of the findings. Registers the core protocol (e.g. in OSF). Ensure the review software is pre-programmed and reviewers and other stakeholders involved are trained in the methodology and process.”

 

- **“CT responsibilities:** Signs-off the final protocol and any amendments. Ensures the protocol is registered in an open-accessible database such as the Open Science Framework and that the review software is pre-programmed, and all stakeholders involved are trained and familiar with the process and review software used.”

            

- On Page 11: “They will refine the systematic review program if updates have been made to the protocol, e.g. updates to the screening eligibility, and/or the extraction program, e.g. activate or deactivate individual sub-domain categories/questions or whole domains to be included in the mapping/data extraction.”

 

- *Page 7, second paragraph:* Is extracting into Microsoft Excel a realistic option if a global team does this across time zones? Should this not be captured in a robust database?

 

- We have clarified this on page 11: “The reviewers will then start the screening (titles and abstracts, followed by full text screening). Followed by data extraction using the pre-defined data extraction template programmed earlier.  Alternatively, if the researchers do not have access to a review software then the screening decisions and the data extraction can be made using Microsoft Excel.  Using a shared Microsoft Excel  platform  will facilitate collaboration and coordination of the review process.

 

- *Page 7, Systematic review software:* Why is the systematic review software included in the study protocol? It would be better to abstract any software components from the study protocol and include a general section outlining what this software should be able to do. Furthermore, this paragraph reads as if the authors intend to use this study protocol solely for themselves rather than provide a generic tool such as PRIMSA, which is a shame, as the main benefit for the wider community is in that.

 

- We have rephrased the paragraph to specify the key features of a systematic review software, see page 12. “If access to a systematic review management software (e.g. DistillerSR, RedCAP, Rayyan, Covidence) s this this will allow processes to run in parallel without the need for handovers, and facilitate  standardisation of extractions through pre-programmed data extraction forms, and   future updates as new evidence emerges. A systematic review software also enables continuous monitoring of progress, rapid identification of issues and facilitate audits and quality checks. A program such as Microsoft Excel can be used as an alternative, but requires more manual administrative management, allocating records for screening and data extraction to reviewers and progress monitoring and manual open-ended extractions.”

 

-  
*Page 7, reporting responsibilities*: Is e-mail an appropriate system to coordinate data? Should this not be done via a database that keeps records of amendments/changes, or via a tool such as GitHub, which also provides change tracking via commits and allows for reviews?

 

- Thank you for this suggestion, we have clarified this on page 12: “The systematic review team/s are responsible for submitting the completed data extraction tables to the coordinating team via secure e-mail or other sharing platform as soon as completed for rapid data sharing. The completed data extraction tables can also be shared using an appropriate, secure systems (e.g. Figshare, GitHub) for open-access data sharing”

 

- *Page 7, research domain categories*: Point 3 should be expanded to include a broader range of data, including the full range of epidemiological parameters and models. Restricting to just human-to-human seems extreme, particularly for vector-borne diseases such as Zika.
- Thank you for this valid comment. Our approach emphases the importance of adopting a modular approach by activating domains that are of essential for the topic studied, including the option to include additional modules. We provide an optional module focused on animal-to-human transmission (see appendix). However, piloting shows that it is not feasible to include more domains to the core set of domains. We have reviewed the potential for including vector-borne transmission, however it is not feasible to expand the RRNA further, and since we include domains focused on effectiveness of prevention and control methods, and implementation factors associated with these, this would require a very different search for vector borne diseases.
- A key eligibility criteria for our RRNA is the inclusion of humans or human samples in the study, as health protection is our focus and to make it manageable. For vector borne disease prevention and control we would risk missing field studies that did not include humans, and thereby risk misinterpreting the evidence landscape. If we changed the search and screening to include vectors only it would create too much noise and make the process infeasible. The existing focus has been informed by expert consultations and piloting and defined to the existing domain categories for relevance for community protection and the early response to outbreaks, and to ensure feasibility and quality.  However, the RRNA concept can be applied to other research questions.

 

- We have clarified how the domain categories were identified on page 5: “The research domain categories has been informed by consultations with experts in emerging infectious diseases research and response, and specialists in One Health, review methodologies, epidemiology, public health, entomologists, veterinarians, social and behavioural sciences. They are focused on evidence relevant to prevention and early outbreak response to protect communities and health workers to improve outcomes.”

 
*Page 8, research domain categories*: Point 4, the risk factor outcomes seem narrow. Why not include other risk factor outcomes, such as death, as well?  

- We have made it explicit that this category includes death, and also long-term sequela, which are all a sign of more severe disease. See page 13 & 23 (table):  Risk factors for infection, severe disease, death, long term-complications

 

- *Page 8, research domain categories*: One category which is missing and would be beneficial to add is Genetics. Understanding evolutionary mutation rates, phylodynamics, and phylogeography is essential. The other missing category is non-pharmaceutical interventions.   

 

- We agree that non-pharmaceutical interventions (NPIs) are important, especially for priority disease outbreaks where there is limited or no other medical countermeasures. These are captured in the Supportive care domain which includes any support to manage symptoms, including e.g. salt fluids, nutrition, psychological support (domain 8). NPIs are also captured in effectiveness of prevention and control methods for infection (domain 3) and factors impacting on implementation of these (domain 9). Further, as part of the domain capturing knowledge, attitude, practice and access (domain 9).  

 

- For genetics, this is partly covered in domain 1 and can be expanded upon in this domain. We have piloted inclusion of phylogenetic studies however, it was not feasible to include, nor deemed immediately essential by the expert. Since the protocol has been designed in a modular approach it can be integrated if deemed relevant, as long as the search terms and eligibility and program is updated accordingly.

 

- We have clarified how the domain categories were identified on page 5: “The research domain categories has been informed by consultations with experts in emerging infectious diseases research and response, and specialists in One Health, review methodologies, epidemiology, public health, entomologists, veterinarians, social and behavioural sciences. They are focused on evidence relevant to prevention and early outbreak response to protect communities and health workers to improve outcomes.”

 

- *Page 8*: The authors say that results will be disseminated through the Pandemic PACT website. Why is that included in the study protocol?
- Thank you for pointing this out. We have deleted references to how we will disseminate our data from the protocol.

 

- *Page 9, Table 6*: The inclusion criteria are limited to human-to-human transmission, which seems too restrictive, especially for vector-borne diseases (e.g., Zika, Dengue). Why are data modelling studies excluded, as they are critical to domain 3? Do the authors intend to exclude theoretical modelling studies that are not fitted to data? Having an asterisk on preprints is ambiguous, and it might **be better to state that during non-outbreak periods, preprints are explicitly excluded, but during outbreaks, they are included.** Also, why are systematic reviews not excluded? If data are extracted and a meta-analysis is performed, the analysis would consist of study results and summary estimates, potentially double-counting some studies but not others, leading to biased estimates. I understand that the aim is to use systematic reviews to map the existing evidence base. Still, the protocol needs to treat systematic reviews more carefully so that they are not included in any meta-analysis or data synthesis.

 

- We acknowledge that modelling studies are relevant in infectious disease preparedness and response. We have reflected on whether to include these, however, due to feasibility and our piloting showing that due to our eligibility criteria being focused on humans or human samples, we would not systematically be able to capture all modelling studies. Researchers can adapt the RRNA concept to include such studies, any modifications will require refining search strategies and eligibility criteria and clearly communicate this to all stakeholders involved. We have clarified this on page 5: By designing it in a modular approach researchers can adapt the concept and modify domains as requires for other purposes, e.g. modify the search strategy and eligibility criteria and target domains focus, add new domains or domain questions.  

 

- We have clarified that systematic reviews are used for informing need and potential for targeting the protocol and that pre-prints will only be considered for rare or new disease with very limited data available and during outbreaks, where pre-prints can provide early evidence relevant to inform strategies during the outbreak.

 

- Page 14 (table): Preprints should mainly be considered for very rare diseases or during outbreaks only.

 

- *Page 9, Table 7*: Step 1: Cases should account for the possibility that some papers may report on multiple outbreaks. Study years would be better captured as study dates, allowing the extraction to capture actual dates if available. Step 1 is suitable for capturing the overall information for the paper; however, it is critical to capture the context for each estimate in each sub-domain, as these may vary (by age group, risk population, location, number of observations/cases, etc.) and could introduce errors in subsequent analysis, particularly if other groups use the extracted data for follow-on work, if not captured accurately.
- We agree that this level of information would be of importance for a systematic reviews and meta-analysis. For the RRNA methodology the aim is to balance rigour with speed, to be able to respond during outbreaks. Further, our piloting shows that this level of information is not consistently presented, and often not available. Hence for the purpose of evidence gaps identification and feasibility we only record study years, and accept that there may be overlap in cases between records, which is acceptable when identifying gaps. Our analysis is descriptive.

 
*Page 10, domain 1*: CFR is the case fatality ratio. This is not a rate because it lacks an associated time unit. If the authors prefer to call it a risk, that would be okay, but it is not a rate. Also, it should be captured if this CFR estimate is a naïve or adjusted estimate (or unspecified).

- Thanks for spotting this, it has been corrected to case fatality ratio. All data is extracted as reported by the study authors.

 

- *Page 10, domain 2*: Do the authors intend to extract the seroprevalence data? This would be of enormous benefit, but it requires much more information in the study protocol, as this is a complex task. Also, this would require the extraction team to be trained and to have sufficient knowledge of serology. This is another example in the manuscript where it is unclear whether the aim is only to highlight gaps or to actually extract data, which is a much more difficult task.
- We agree that extracting seroprevalence data is beneficial, but we also acknowledge that it is complex and out of scope for the purpose of the RRNA. We are extracting /mapping key data such as study design, study year, setting, populations groups to show where there are gaps in data and provide a repository of existing studies. Hence, if there are a sufficient number of studies identified on any of the domain topics, these can readily be pivoted to a full systematic review if needed and resources are available.

 
*Page 10, domain 3*: Why is only the incubation period being extracted/recorded? This should extend to all delays, including the serial interval, the onset-to-hospitalisation interval, etc. Also, it would be great to capture whether only sample summary statistics (mean, median, min, max, etc.) are reported or whether distributions are fitted.

- Based on our piloting of the protocol we have found that it is not systematically reported for us to meaningfully report on it. The RRNA is developed for emerging infectious diseases where the evidence base and knowledge is limited. For these diseases the published evidence is generally also limited by study design, numbers included, and quality of reporting. Many studies are cross-sectional reporting sparse data from healthcare centres in resource limited settings, often lacking in reporting even of basic demographics. We collect and extract available data in the format available, whether this is mean or median or just an age range. Due to the nature of the publication landscape for these disease, the RRNA is focused on essential data mapping. If there were a large number of records identified, these can be pivoted to more in-depth extraction due to the benefit of the adaptive protocol.

 

- *Page 10, domain 3: *It would be good to capture reproduction number estimates. More generally, several transmission parameters, such as overdispersion, should be included in this domain to enable infectious disease epidemiologists to build models.

Can be targeted depending on the disease—resource and quality

- Based on our piloting of the protocol we have found that it is not systematically reported for us to meaningfully report on it. See previous comment.

 

- *Page 12, search terms*: Thank you for including the search terms. In an outbreak situation, this is only possible since the last RRNA update, as otherwise one would get too many titles and abstracts to screen (e.g., for Ebola or SARS-CoV-2, this would exceed 10,000 papers if the databases are searched since inception).
- We agree with this observation, hence we have specified that the search strategy and inclusion dates should be defined for each disease and context. Further that the protocol is developed for disease where the evidence base is limited. Ebola and SARS-CoV-2 is not fit for a full RRNA. For other re-emerging diseases which has been researched over time, it may be relevant to limit the search strategy to ensure inclusion of up-to-date data only, whereas for rare diseases with sporadic outbreaks only we recommend to include data from first detection of the disease.
- *Page 13, Screening*: Has the use of Google Translate been tested and validated for inclusion in the study protocol? Is there a risk that the translation introduces errors?
- Based on our piloting where we have compared Google translate and checked the translations with reviewers fluent in the language, which has shown that for many common languages it is reliable enough for the purpose of the RRNA. However, it does not work for all languages, in which case a reviewers with the language skills needs to be identified, or the record excluded from the data extraction and instead listed with a full bibliography in the appendix. This has been updated on page 18.

*Page 13*: A second reviewer will check up to 70% of the data – this is huge. Why can’t there be some double extraction at the beginning until the extractors converge to a consistent extraction? Otherwise, this may not be feasible given the time frame.  

- In our experience we find that this is feasible, since the data extraction is focused on mapping key variables, rather than open ended extractions. Depending on the volume of articles identified and the need for rapid results, the proportion can be reduced, that is where we state up to 70%, we have further refined this to 30 to 70% of the data depending on volume of records, needs and resources. For rare diseases this is feasible, for high volume RRNAs it can be reduced as required. The data can be checked in the review software or in the exported Excel data file during the data cleaning and analysis process.  This has been clarified on page 18: “A second reviewer will check a minimum of 30% and up to 70% of the data, depending of volume of records, need and resources.”

 

- *Page 14, full data extraction*: This would be nice, but how feasible is that unless the team is already in place, fully trained, has all the tools at their disposal and only needs to update since the last entry into the database? Even in the most minimal form, this would be a considerable effort, especially if up to 70% of extracted data is double-checked.
- *Page 14, Definitions*: Please be more specific about the case definition.
- We have specified these further, see page 14.
- *Page 14, Definitions*: The age ranges should be more flexible, as each paper will use its own definition.
- We have included wide age groups definitions to allow for that flexibility, and again this can be adapted as required/needed for each RRNA. For emerging infectious diseases we are aware that certain population groups have not been included in studies in the past for different reasons, even though they are often of higher risk of severe disease. Therefore, it is important to include specific age groups especially for children, where the risk factors are different depending on age, e.g. neonates/infants, young children versus older children. If aiming to collect these but the data is not there, they can be aggregated at the analysis stage. We have clarified this on page 14: For age-groups, it is of relevance for identifying evidence-gaps to collect data on different risk populations.  If the data is not available, age groups (e.g. neonates/infants can be aggregated at the analysis stage.

 
*Page 14, data analysis and dissemination*: The authors state that they will provide the analysis code. Why is this included in a study protocol? Would it not be better to set out the requirements and standards for the analysis? Also, several tools already exist for this (epiverse, epireview) and are in use.

- Thank you, we have deleted this statement from the protocol.

- *Page 14, data analysis and dissemination*: In the second paragraph, the authors provide a helpful discussion of what should be done during preparedness time versus during an outbreak. It may be helpful to consider existing work in this area ( https://mrc-ide.github.io/epireview/) as well as broader WHO initiatives, such as grEPI.
- Thank you for this suggestion. We have kept the text general, as we acknowledge that different groups might adapt the protocol for different purposes. For the purpose of the RRNA we are identifying and including published evidence in manuscripts and reports as defined in the eligibility criteria. Data repositories are out of scope for the RRNA. The data collected can be deposited in repositories in adherence with good data sharing practices.
- *Page 15, RRNA updates*: It would be helpful if the authors could expand this section substantially. This is non-trivial, and setting this out in the protocol would be helpful. A good example is how database entries should be updated between preprints and the final published paper.
- We have expanded on the updating of the RRNA as described in earlier comment, to clarify that this can be done as new evidence is generated e.g. during or after outbreaks. Since the RRNA is focused on identifying gaps in the evidence-base and providing a method that can be implemented systematically and rapidly in response to outbreaks/when need arises, we can allow some duplication of data, since we are identifying gaps in the evidence base, not performing a systematic review.
- We have also clarified that we will only include pre-prints if deemed relevant, e.g. during outbreaks when key data may be published first in a rapid pre-print, and waiting for the peer-reviewed publication would not make the data available in a timely manner to inform response strategies
- If the RRNA is updated subsequently the pre-print may be removed if the subsequent publication is available – updated on page 28: “The RRNAs can be updated when new key evidence emerges, e.g. during and after outbreaks, or implementation of new therapeutic or vaccine trials. Such update could include updating the reference library to include newly identified studies and revising aggregate counts accordingly.  If pre-prints were included in the earlier RRNA, these can be updated with the subsequent publication if available.”

 

- In the discussion, the authors raise the essential point of collaborative partnerships, which will be critical to this type of work. However, the key question is who convenes and funds this (not to be answered by this paper). Rapid and robust protocol that could be done quickly. Rapidly and can pivot to proper systematic reviews. During non-emergency periods, systematic reviews with meta-analysis and data synthesis should be strongly preferred as the gold standard. These reviews should be designed to be updated, and the underlying extracted data should be publicly available. This paper fills a critical gap in the literature on how these datasets can be rapidly updated to support outbreak response. Overall, this is an excellent contribution to the literature, but it would benefit from a more precise separation of the study protocol from tools such as the systematic review software, reference manager, or dissemination tools. For the study protocol itself, it would be helpful to separate the use case of mapping existing data from that of extracting it, which is a more complex task.
